# In situ tunable, room-temperature polariton condensation in individual states of a 1D topological lattice

**DOI:** 10.1126/sciadv.adt8645

**Published:** 2025-05-28

**Authors:** Ioannis Georgakilas, Rafał Mirek, Darius Urbonas, Michael Forster, Ullrich Scherf, Rainer F. Mahrt, Thilo Stöferle

**Affiliations:** ^1^ IBM Research Europe – Zurich, Säumerstrasse 4, 8803 Rüschlikon, Switzerland.; ^2^ Institute of Quantum Electronics, ETH Zurich, Auguste-Piccard-Hof 1, 8093 Zürich, Switzerland.; ^3^ Macromolecular Chemistry Group and Wuppertal Center for Smart Materials & Systems (CM@S), Bergische Universität Wuppertal, Gaußstraße 20, 42119 Wuppertal, Germany.

## Abstract

Exciton-polariton microcavity arrays have emerged as a promising semiconductor-based platform for analog simulations of model Hamiltonians and topological effects. To study a variety of Hamiltonians and investigate their properties, it is essential to have highly configurable and easily engineerable structures with low disorder. Here, we demonstrate in situ tunable, room-temperature polariton condensation in individual states of a Su-Schrieffer-Heeger topological lattice by using an open-cavity configuration with an organic polymer layer. Changing the cavity length in combination with vibron-mediated relaxation in the polymer allows us to achieve selective polariton condensation into different states of the band structure, unveiled by nonlinear emission, linewidth narrowing, energy blue shift, and extended macroscopic coherence. Furthermore, we engineer the bandgap and the edge state localization by adjusting the interaction between adjacent lattice sites. Our results demonstrate the versatility and accuracy of the platform for the investigation of quantum fluids in complex potential landscapes and topological effects at room temperature.

## Teaser

Exciton-polaritons allow for taking accurate pictures of the wave function of quantum fluids in tunable topological lattices.

## INTRODUCTION

Cavity exciton-polaritons (polaritons in what follows) are hybrid light-matter quasiparticles resulting from strong coupling between an optical microcavity mode and an excitonic transition in a semiconductor (*1*). Polaritons behave as composite bosons that, when reaching a critical density, can undergo nonequilibrium Bose-Einstein condensation (*2*), marked by the appearance of macroscopic coherence and superfluidity. Polariton condensates have been realized using a variety of material systems, originally in II-VI (*3*) and III-V (*4*) compound semiconductor heterostructures and, more recently, in materials like organic (*5*, *6*), perovskite (*7*), and two-dimensional (2D) (*8*) semiconductors, providing simplified processing and enabling room-temperature operation. Polariton condensates have been instrumental to study fundamental phenomena such as quantized vortex formation (*9*), superfluidity (*10*), and Kardar-Parisi-Zhang physics (*11*) but also allowed applications in photonic devices, including polariton lasers (*12*) and all-optical logic (*13*–*15*).

Arrays of coupled polariton condensates are an increasingly popular platform for analog simulations of Hamiltonians, drawing inspiration from the field of ultracold atomic gases. By using established nanofabrication techniques, polariton lattices can be created where the hybrid light-matter nature of polariton condensates allows for shaping and controlling the photonic part of their wave function, while interparticle interactions are supported through their matter part. Moreover, spectroscopic techniques combined with reciprocal- and real-space imaging enable direct measurement of the lattice’s band structure and the amplitude of the wave function (*16*). Motivated by this, a variety of 1D (*17*–*20*) and 2D (*21*–*25*) polariton lattices have been realized, allowing for studying a plethora of physical effects such as topologically protected lasing (*26*, *27*), flat band formation (*28*–*30*), polaritonic graphene (*31*), and spin frustration (*32*).

**Fig. 1.**
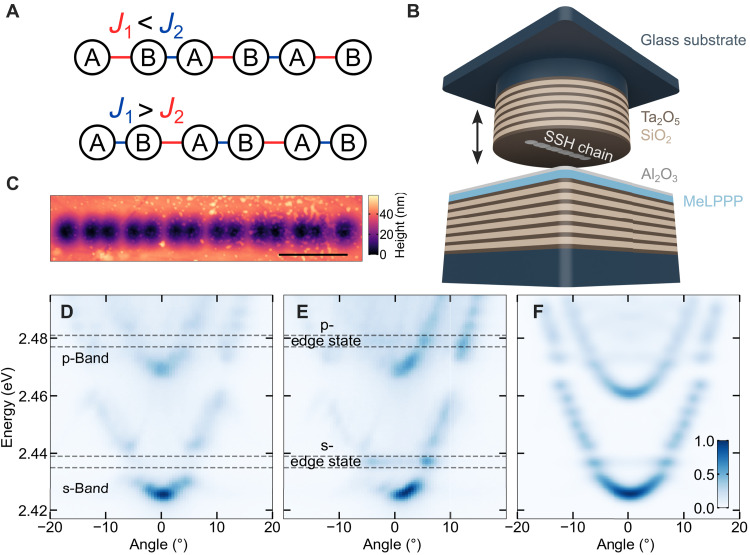
Polaritons in an SSH chain. (**A**) Sketches of the topologically nontrivial (top) and trivial (bottom) SSH configurations. The large spacing and red color indicate small effective coupling, whereas the small spacing and blue color indicate large effective coupling. (**B**) Illustration of the used tunable cavity composed of two separate halves. The vertical black arrow indicates that the distance between the two halves can be tuned. The bottom half is composed of the organic layer (blue color) on top of a DBR (alternating layers). The top part consists of a patterned DBR where the gray-colored structure indicates the patterned SSH chain. (**C**) Atomic force microscopy image of the studied structure. The scale bar equals 3 μm. (**D** and **E**) Angle-resolved photoluminescence measurements of the SSH chain performed when exciting at the middle (D) and at the edge (E) of the structure. The band structure is composed of s- and p-like bands (D) and two SSH gaps. Measuring the structure’s edge reveals the topological edge states inside the SSH gaps (E). Two dashed lines indicate the region of the SSH gaps and clearly visualize the absence of the edge states in (D) and their appearance in (E). (**F**) Simulated band structure of the SSH chain. The angle of the photoluminescence is along the lattice structure.

In this work, we investigate a polariton Su-Schrieffer-Heeger (SSH) chain, i.e., a 1D lattice composed of adjacent sites with two alternating coupling strengths (*33*). This model was originally established to mimic the alternating single and double bonds in polyacetylene (*34*) and, more recently, has been implemented as a basic model supporting topologically protected states. Figure 1A displays the two distinct configurations of a finite-length SSH chain (*35*): (i) the so-called topologically “trivial” configuration (the chain starts and ends with the strong bond), which does not support topological states, and (ii) the topologically “nontrivial” configuration (the chain starts and ends with the weak bond), which induces the formation of topological edge states inside a forbidden energy gap (SSH gap in what follows) arising from the chain’s staggered nature.

Many previous studies were based on monolithic cavities, where the system was engineered such that polaritons condense in a specific, desired state (*36*–*40*). In the present work, we exploit an in situ tunable cavity configuration, in combination with vibron-mediated polariton scattering (*14*, *41*), allowing for achieving selective polariton condensation in different topologically trivial and nontrivial states. In comparison to previous experiments claiming selective condensation (*17*, *24*, *36*), in this work, we provide a comprehensive “recipe” for tunable condensation, and we demonstrate simultaneously highly selective, single-mode condensation with a substantial tuning range, in situ at the same lattice structure. Therefore, within the very same SSH chain, we can observe the formation of two distinct topological edge state condensates, originating from two bands with different symmetries and separated by about 40 meV. Furthermore, we demonstrate precise control over the size and energy of the SSH gap and the localization of the topological edge states and compare it to ab initio numerical calculations. We show that a larger contrast between the two couplings leads to increased SSH gaps and to tighter confined edge states, which has been theoretically predicted but not experimentally realized. This led to the observation of the highest reported SSH gaps (>10 meV) (*26*, *27*, *37*–*40*), which is crucial for the robustness of topologically protected lasers. Our results showcase the potential and the accuracy of this highly tunable and easy-to-engineer platform for realizing a variety of single-mode extended coherent states, which are essential for conducting room-temperature analog simulations (*42*), investigating topological effects (*43*–*45*), and performing optical computing (*46*).

## RESULTS

We use a tunable open-cavity setup, which is composed of two separate halves, as illustrated in Fig. 1B. The fabrication and setup are detailed in Materials and Methods. In short, the bottom cavity half is composed of a glass substrate, coated with a distributed Bragg reflector (DBR) and, on top of it, a thin layer of methyl-substituted ladder-type polymer (MeLPPP) with additional encapsulation. Aside from the vibron-mediated polariton scattering mechanism, we selected this organic material for our room-temperature experiments over perovskites or 2D materials because it provides a combination of advantages: high stability, good homogeneity to support extended polariton lattice condensation, and the potential to perform single-shot measurements (*47*). The top cavity half consists of a DBR deposited on a glass substrate in which arrays of Gaussian-shaped deformations have been patterned, serving as potential wells for the polaritons where alternating center-to-center spacings effectively form an SSH chain. Each Gaussian deformation constitutes a single lattice site, which induces lateral confinement to the polariton wave function, resulting in the formation of a set of on-site discrete energy states (*48*). In addition, engineering the spatial overlap of neighboring sites allows for controlling the coupling strength between them (*49*). The cavity halves are mounted on separate nanopositioning stages for *XYZ* translation, enabling to tune the cavity length and, therefore, precisely control the energy of the cavity resonance. As the SSH band structure makes precise, direct determination of the light-matter coupling strength with coupled-oscillator models impractical on the lattice structure, we collect white light transmission spectra at an unstructured spatial position directly next to the studied lattice structure, where we measured a Rabi splitting of 2Ω = 144 meV (see fig. S1). The detuning of the cavity from the exciton is about −200 meV, corresponding to an excitonic fraction of ~11.5% in the lower polariton branch, which is in line with a dominantly photonic regime being quite common for room-temperature polariton condensates (*6*, *27*, *50*–*52*).

### Polaritons in an SSH chain

We study a topologically nontrivial chain configuration, composed of 14 lattice sites, with 0.72- and 1.08-μm spacings for the strong and weak bonds, respectively, resulting in effective couplings *J*_1_ = 9.2 meV and *J*_2_ = 4.6 meV (see the Supplementary Materials). The structural characterization of the lattice and the measurements revealing its topological nature are reported in Fig. 1 (C to F). An atomic force microscopy measurement of the structure (Fig. 1C) shows a total chain length of ~14 μm with a depth of ~40 nm and a full width at half maximum (FWHM) of ~1.1 μm for each Gaussian deformation. We populate the band structure of the lattice with polaritons by exciting the sample below the polariton condensation threshold with an off-resonant continuous-wave laser. Exciting locally (two to three lattice sites) and detecting the angle-resolved emission at the center of the lattice allow us to observe the well-resolved band structure composed of s- and p-like bands and the formation of energetic bandgaps of the order of 10 meV (Fig. 1D). s- and p-SSH gaps that open because of the bond alternation are indicated with the dashed lines. By repeating the measurement at the edge of the chain, the band structure exhibits two nondispersive energy states that appear inside the SSH gaps (Fig. 1E), indicating the formation of topological edge states within the polariton lattice’s band structure. The observed band structure is characteristic for a linear SSH chain and can be theoretically reproduced by solving the Schrödinger equation for the full potential in the real space (Fig. 1F) (see the Supplementary Materials).

### Selective polariton condensation in different lattice modes

**Fig. 2.**
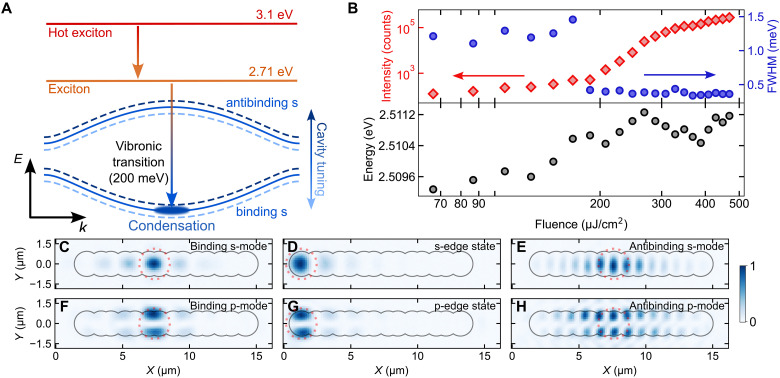
Tunable polariton condensation. (**A**) Sketch of the vibron-mediated polariton condensation process. We create hot excitons (red) using an excitation photon energy of 3.1 eV by driving the system nonresonantly. Through internal conversion, the hot excitons relax and form the exciton reservoir (orange) at 2.71 eV. The displayed dispersion (blue) is typical for the folded s-band of the SSH chain. Tuning the cavity length shifts the dispersion in energy (dashed lines), bringing different parts of it in resonance with the 200-meV vibronic transition and therefore inducing efficient polariton condensation in different lattice modes. (**B**) Excitation fluence–dependent measurements of the emission intensity (top panel, red diamonds, left axes), FWHM (top panel, blue dots, right axes), and blue shift (bottom panel). (**C** to **H**) Real-space images of different topologically nontrivial (D and G) and topologically trivial (C, E, F, and H) lattice modes in which one can selectively condense. An overlaid gray sketch of the lattice’s outline allows for referencing the different emission patterns with respect to the lattice sites. The excitation spot position and dimension are marked with red dashed circles. Each real-space image is normalized, and the emission intensity is mapped by the color bar at the very right. All the condensates were measured slightly above the threshold.

Next, we studied the emission of the structure above the polariton condensation threshold, as displayed in Fig. 2. Upon nonresonant excitation of the organic polymer MeLPPP, hot excitons are formed, which relax predominantly through internal conversion, forming an excitonic reservoir. A single-step relaxation scheme including a molecular vibronic excitation enables efficient population of polariton states one vibron energy below the excitonic reservoir, avoiding inefficient multistep relaxation processes (*53*, *54*). Therefore, in our organic polymer, polariton condensation occurs efficiently when the detuning between the polaritonic mode and the exciton reservoir matches a strong vibronic transition (in our material, 200 meV) (see Fig. 2A and fig. S2). When exciting locally (two to three sites) at the lattice’s edge, polariton condensation in the s-band topological edge state has been observed, displaying the expected condensate signatures of nonlinear increase in the emission intensity, FWHM narrowing, and energy blue shift, with a condensation threshold between 100 and 200 μJ cm^−2^ (Fig. 2B). The value of the observed threshold fluence is consistent with the reported polariton condensation threshold of 130 μJ cm^−2^ in the ground state of a single Gaussian-shaped cavity with the same active layer (*48*). By tuning the cavity length to energetically shift the band structure and using the vibron-mediated condensation process, we can induce polariton condensation in different topologically nontrivial and topologically trivial states of our structure (Fig. 2, C to H, and fig. S3). The two experimental parameters needed to adjust for mode-selective condensation are the cavity length and the position of excitation. Exciting at the edge of the lattice allows for selectively condensing into either the s- or p-edge states (Fig. 2, D and G), whereas exciting at the center of the chain allows for changing between the binding and antibinding s- and p-band (trivial) bulk modes (Fig. 2, C, E, F, and H) by adjusting the resonance.

**Fig. 3.**
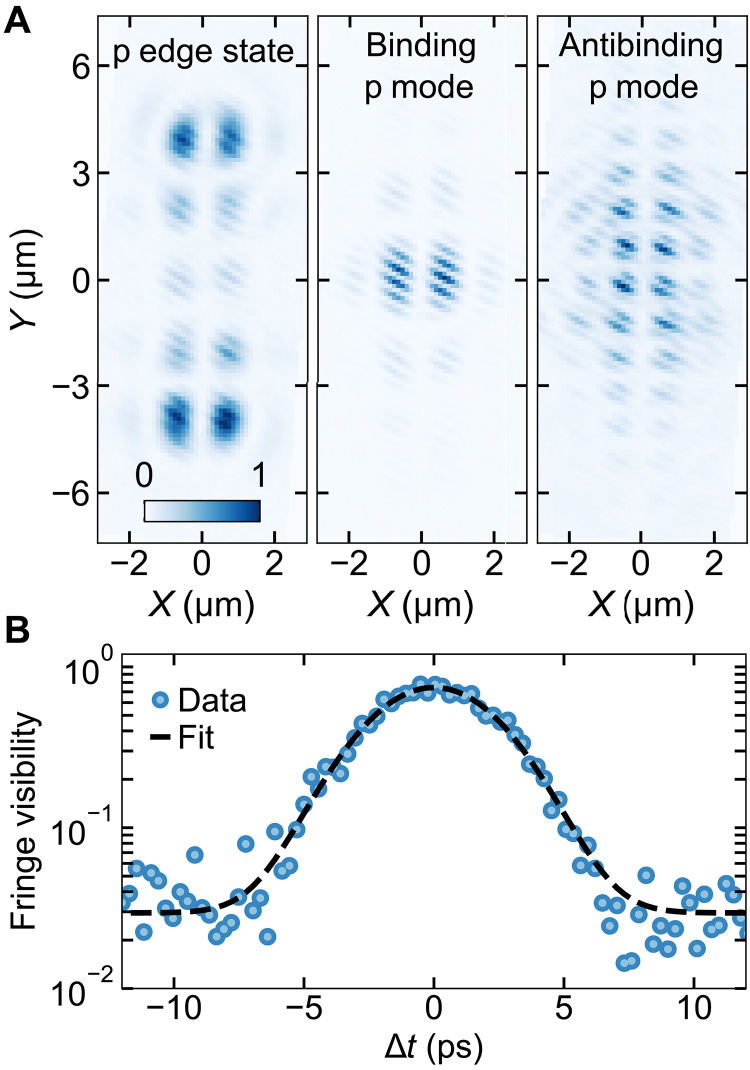
Condensate spatial and temporal first-order coherence. (**A**) Interferometric measurements of the three p lattice mode condensates. For each measurement, the interference fringes extend through the whole condensate. Each image has normalized intensity, which is described by the color bar inset at the bottom of the edge state image. All the condensates were measured slightly above the threshold. (**B**) Measurement of the temporal coherence of the binding p mode condensate. The fringe visibility at each time delay Δ*t* was determined by performing a Fourier transform of the images, isolating the signal at the spatial frequency of the fringes, and normalizing it by the total signal in the Fourier-transformed image. This calculated visibility was then calibrated by comparing to the zero-time-delay visibility, which was directly extracted from the fringe minima and maxima in the real-space image. The dashed black line represents a Gaussian fit to the data with an FWHM of 5.8 ps.

As extended macroscopic coherence is a hallmark of polariton condensation, we used a Michelson interferometer setup with a retroreflector that spatially inverts the emission image in one arm to study the spatial first-order coherence of the polariton condensates. The results are presented for the edge state mode and the binding and antibinding modes of the p-band manifold in Fig. 3. The interferograms show that the condensates exhibit spatial coherence extending over nearly the whole structure (Fig. 3A). The highest intensity of the condensate appears at the edges in the case of the edge state (Fig. 3A, left panel) and at the center for the bulk modes (Fig. 3A, middle and right panels) because of local excitation at the edge and the middle of the structure, respectively. Subsequently, tuning the length of one of the two interferometer arms, which changes the effective time delay Δ*t*, allows for measuring the temporal behavior of the condensate coherence by extracting the fringe visibility of the interferogram, resulting in a Gaussian autocorrelation function with an FWHM of 5.8 ps (Fig. 3B). Such a Gaussian envelope for the phase coherence can be attributed to number fluctuations and polariton interactions (*55*). The observed coherence time is an order of magnitude longer than the polariton lifetime in the system and is comparable to the excitation pulse duration, which effectively determines the coherence time of the driven-dissipative polariton condensate.

### SSH gap and edge state engineering

**Fig. 4.**
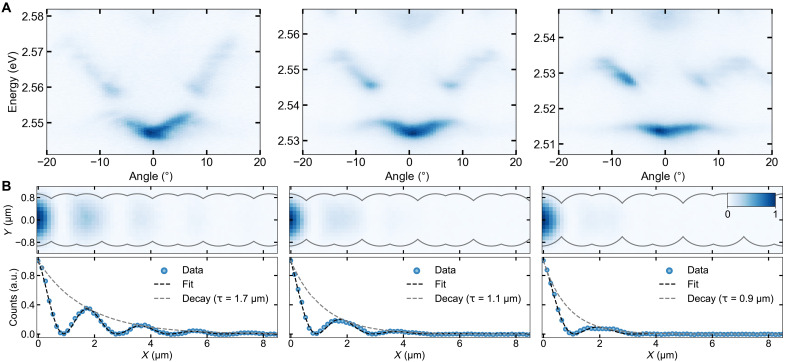
SSH gap and topological edge state engineering. (**A**) Angle-resolved photoluminescence measurements of three SSH lattices with varying weak bonds (from left to right: 4.6, 3.2, and 2.0 meV), showing the evolution of the bands and the bandgap of an SSH chain in a topologically trivial configuration. (**B**) The top panels show the real-space mode profiles of the condensed s-band edge states overlaid with the lattice’s outline for three nontrivial structures with matching bandgaps as in (A), while the bottom panels show the fitted line profiles of the three edge states with their respective calculated decay lengths (intensity counts were integrated over the vertical real-space image axis). All three edge states were measured slightly above the threshold and at exciton-photon detunings, which resulted in all three edge states condensing at the same energy ~2.55 eV. a.u., arbitrary units. All the images in (A) and (B) are normalized and are described by the color bar inset in the top right part of (B).

In view of an analog polariton simulator, the developed structure should be easily engineerable to realize varying parameters of the Hamiltonian under study. By measuring three separate chains with different coupling strengths, we assess the impact of the interaction between sites on the topological features and the band structure of the lattice, as shown in Fig. 4. The three investigated chains share common strong bond coupling (*J*_1_ = 9.2 meV), while they have different weak bonds (*J*_2_ = 4.6, 3.2, and 2.0 meV). The angle-resolved measurements show that for larger site spacing (weaker coupling), corresponding to an enhanced coupling contrast between weak and strong bonds, the SSH gap becomes larger, in line with theory predictions (Fig. 4A) (*35*). Moreover, by studying the real-space emission of the condensed edge state in the three chains with different bandgap sizes, we can demonstrate the effect of the bandgap on the localization of the edge state (Fig. 4B). As expected from theory [see the Supplementary Materials and (*35*)], the larger the SSH bandgap is, the more isolated is the edge state with respect to the bulk modes, leading to tighter spatial localization at the edge of the lattice (Fig. 4B, top panels) with a dependence of τ=1ln(J1J2) obtained from the tight-binding model. In addition, by fitting the mode profiles with sinus-squared modulation with an exponentially decaying envelope, we extract an exponential decay length τ for each state (Fig. 4B, bottom panels).

**Fig. 5.**
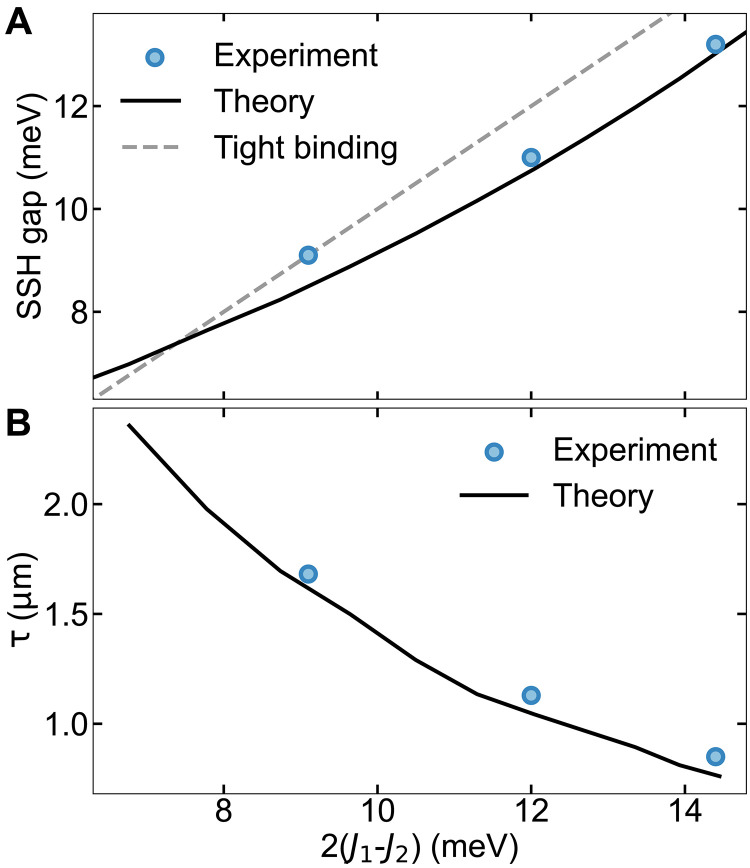
SSH gap and edge state decay versus 2(*J*_1_-*J*_2_). (**A**) Plotted experimental (blue dots) and simulated (black line) SSH gap width versus 2(*J*_1_-*J*_2_). The observed SSH gap’s slightly nonlinear increase matches well with the numerical simulations. (**B**) Extracted from the fit of the spatial images (Fig. 4), experimental (blue dots) and simulated (black line) decay lengths versus 2(*J*_1_-*J*_2_). Experiment and theory agree well, and both illustrate the tighter confinement of the topological edge state when it resides in a larger SSH gap.

Figure 5 summarizes the direct effect of the engineered coupling to the SSH gap variation and the edge state localization and compares the experimental results to the numerical simulations (for additional details, see the Supplementary Materials). The measured width of the SSH gap (Fig. 5A, blue points) increases in a slightly nonlinear way versus the difference between the two couplings *J*_1_ and *J*_2_. This increasing trend matches well the simulations, where we solve the Schrödinger equation (black line), in contrast to a perfectly linear trend predicted by a simple tight-binding model (gray dashed line) (*35*). The experimentally obtained decay lengths of the three different edge states (Fig. 5B, blue points) are also plotted versus the difference between the two couplings. The larger SSH gap results in more confined edge states, again observing a good match between experiment and simulations.

## DISCUSSION

We have presented a highly tunable platform allowing for studying organic, room-temperature polariton condensation in extended topological lattices. The topological edge states have been implemented by a 1D cavity array, mapping to an SSH chain model. Measurements below the condensation threshold yield a band structure with s- and p-band manifolds, which, when excited locally at the edge of the chain, displayed the formation of two edge states, confirming the topologically nontrivial nature of the structure. We demonstrated polariton condensation in the s-edge state by driving the system above the threshold while pumping spatially at the edge of the structure. Vibron-assisted relaxation, together with the tunable cavity configuration, allowed us to selectively condense polaritons in different lattice modes, showing polariton condensation in two distinct topological edge states originating from bands with different symmetries as well as in different bulk states.

All condensates exhibited spatially and temporally extended coherence, revealed by interferometric measurements using a Michelson interferometer. Furthermore, we engineered the SSH gap and therefore altered the edge state localization by controlling the coupling strength within the subunits of the SSH chain. The formation of a larger gap for enhanced coupling-strength contrast results in a tighter confined edge state. The fact that our experiments accurately match first-principles calculations emphasizes the quality and faithfulness of the analog simulations. Our results furthermore demonstrate that photonic and excitonic disorder can be kept sufficiently low so that no localization or fragmentation occur in this 1D lattice. Hence, this manifests the high degree of engineerability and tunability of our system, which holds great promise for exploring complex potential landscapes and topological properties within the realm of photonics as well as many-body physics with bosonic quantum fluids under ambient conditions.

## MATERIALS AND METHODS

### Experimental design

#### Fabrication

We use a tunable, open-cavity setup, with a resonator composed of two separate halves. Both “half cavities” are mounted on *XYZ* nanopositioning stages to tune the distance between them and have additional tilting degrees of freedom to allow for parallel alignment. The “top” half has been fabricated by performing optical lithography and wet etching with concentrated HF to create a ∼30-μm-tall and ∼200-μm-wide mesa structure in the center of a glass substrate (1 cm by 1 cm). The mesa reduces the effective surface area of the two approaching cavity halves, therefore strongly decreasing the sensitivity to particle contamination inside the tunable resonator, allowing the two parts to approach routinely on a hundred nanometer scale. On top of the mesa’s surface, we used focused ion beam milling to pattern several 1D arrays of spatially overlapped Gaussian deformations with alternating center-to-center spacings, equivalent to SSH chains. By means of ion beam deposition, 6.5 quarter-wave layer pairs of Ta_2_O_5_/SiO_2_ have been deposited to fabricate a DBR, which retains the morphology of the underlying substrate/pattern. For the “bottom” half, a 35-nm-thick MeLPPP (*56*) [*M*_n_ (number-averaged molecular weight) = 31,500; *M*_w_ (weight-averaged molecular weight) = 79,000] film is deposited by spin-coating from a 1 wt % toluene solution on a flat glass substrate comprising another DBR mirror with 9.5 layer pairs with an additional 20-nm SiO_2_ spacer. The polymer film is protected from photodegradation by encapsulation with a 20-nm-thick Al_2_O_3_ layer, deposited by means of an electron beam evaporator. In addition, the encapsulation layer minimizes the possibility of transferring contaminants between the two cavity halves when they come in contact.

#### Optical characterization

Measurements below the condensation threshold have been conducted with a continuous-wave, 405-nm laser diode, coupled to a single-mode fiber. To drive the system to the condensation regime, a frequency-doubled, amplified Ti:sapphire laser at 400 nm with a 1-kHz repetition rate and ~150-fs pulse duration was coupled into a single-mode photonic crystal fiber. The coupling to the fiber results in a stretching of the pulses to several picoseconds. The excitation was focused on the sample by a 100× microscope objective with a numerical aperture of 0.5, resulting in beam sizes of around 1.4 to 1.6 μm and nearly Gaussian beam profiles. The same objective is used for collecting the signal corresponding to the different mode profiles and condensate threshold measurement in Fig. 2. For the angle-resolved and interferometric measurements, we collect the light exiting from the bottom cavity half with a 20× objective with a numerical aperture of 0.5. For the angle-resolved dispersion imaging, the signal is sent to the front entrance of a 0.5-m-long monochromator (with 300 and 1800 lines/mm gratings) coupled to a liquid nitrogen–cooled camera detector, with the lattice structures aligned parallel to the slit. For the first-order coherence measurements in Fig. 3, the signal is instead sent to a Michelson interferometer with a corner retroreflector in the adjustable arm path.
