# Supplementary material for: In situ tunable, room-temperature polariton condensation in individual states of a 1D topological lattice
Source: Sci Adv. 2025 May 28;11(22):eadt8645. doi: 10.1126/sciadv.adt8645 (PMC12118546; doi:10.1126/sciadv.adt8645)
Supplement: Supplementary file 1 — Supplementary Text Figs. S1 to S11 [file sciadv.adt8645_sm.pdf]

Science Advances
AAAS

Supplementary Materials for

In situ tunable, room-temperature polariton condensation in individual states of a 1D topological lattice

Ioannis Georgakilas *et al.*

Corresponding author: Thilo Stöferle, tof@zurich.ibm.com

Sci. Adv. **11**, eadt8645 (2025)
DOI: 10.1126/sciadv.adt8645

This PDF file includes:

Supplementary Text
Figs. S1 to S11

Supplementary Text

Theoretical model – two-dimensional Schrödinger equation

To describe the observed effects, we perform a first-principles calculation using an effective Hamiltonian for 2D massive particles:

$$\hat{H} = \frac{\hbar^2 \mathbf{k}^2}{2m_{\text{eff}}} + V(\mathbf{r})$$

where $\mathbf{k}$ corresponds to the wavevector in 2D momentum space, m_{eff} is the effective mass and $V(\mathbf{r})$ is the 2D potential formed by the SSH chain of Gaussian-shaped deformations. We diagonalize the Hamiltonian to obtain the eigenmodes and eigenvalues. The dispersion relations were obtained by calculating the Fourier transform of the eigenmodes and assigning them a Gaussian width, matching the experimentally observed value of around 4 meV.

Demonstration of the strong coupling regime

To determine that our system is in the strong coupling regime we probed the sample at a position next to our lattice structure using white light excitation and recorded the transmission spectra for different cavity lengths. As displayed in Fig. S1A, tuning the energy of the cavity resonance around the exciton leads to a clear anti-crossing behavior, characteristic of the strong coupling regime. By fitting the experimental data with a two-coupled-oscillator model we extracted a Rabi splitting of $2\Omega = 144$ meV. For determining the length of our cavity, we performed a transfer matrix simulation of our system and compared it to the extracted polariton dispersion from the experiment as shown in Fig. S1B. As evidenced by the Fig. S1, between consecutive spectra the cavity length is changing by ~3 nm and the energy of the photon mode by ~3 meV, which in practice means that we can control the cavity length with nanometer precision. This becomes possible by bringing the two cavity halves in contact, where an effective lever arm is created, translating the *Z*-stage movement, which would otherwise have coarser resolution, to quasi-linear nanometer change of the cavity length. Furthermore, this contact point strongly suppresses vibrations, allowing to achieve measured condensate linewidths down to 0.3 meV even in a noisy environment.

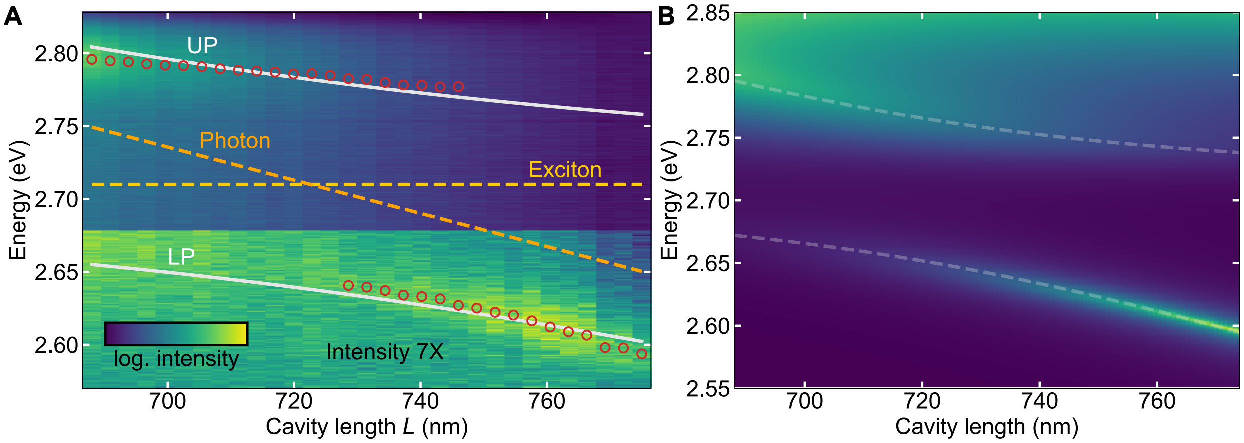

Fig. S1. Emergence of the strong coupling and polariton formation. (**A**) White light transmission spectra as a function of the cavity length. The open red circles indicate the extracted peaks from the experimental data at each cavity length. The two solid white lines show the fitted upper polariton (UP) and lower polariton (LP) dispersions. The yellow dashed line indicates the exciton resonance at 2.71 eV and the orange dashed line the fitted purely photonic cavity dispersion. The signal within the area containing the LP branch is artificially enhanced by multiplying seven times the intensity. (**B**) Transfer matrix simulation of transmission at different cavity lengths matching the experiment overlapped with the fitted polariton dispersion (white dashed lines). The color bar in (**A**) describes both images.

<u>Optical properties of the organic polymer</u>

Fig. S2 shows photoluminescence (PL, excited at 3.1 eV) and photoluminescence excitation (PLE, detected at 2.51 eV) measurements on the utilized organic active layer outside of the cavity structure. The exciton peak is observed in the PLE at ~2.71 eV and the two vibronic replicas at higher energies with about 200 meV spacing.

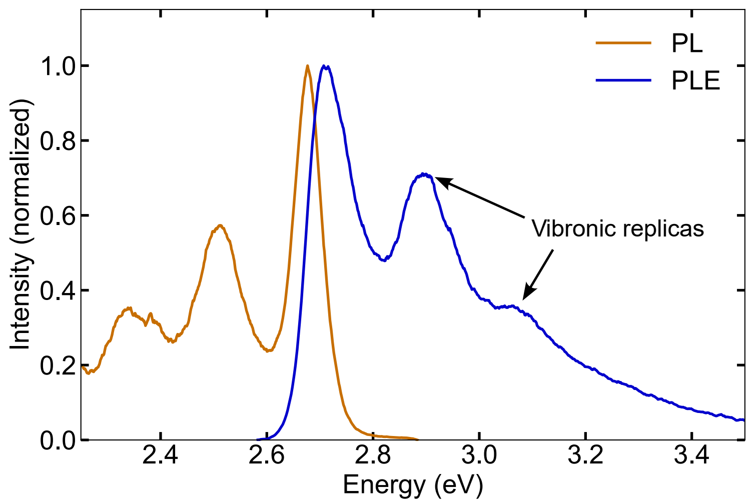

Fig. S2. Optical properties of the organic polymer. PL (orange) and PLE (blue) spectra of the organic polymer MeLPPP. The two arrows indicate the two vibronic replica peaks of the excitonic transition.

Selective polariton condensation in Fourier space

Fig. S3 demonstrates three examples of selective polariton condensation in three different parts of the band structure. By tuning the cavity length, the band structure has been shifted energetically for each different measurement in order to achieve polariton condensation in the desired state. To probe the band structure below condensation threshold we measured the angle-resolved transmission spectrum under white light excitation with a beam size of ∼10 µm (left panels). Consecutively, using pulsed optical excitation we drove the system above threshold (right panels), resulting in polariton condensation in states with well-defined, non-zero *k*-values (angles), characteristic for the respective band structure.

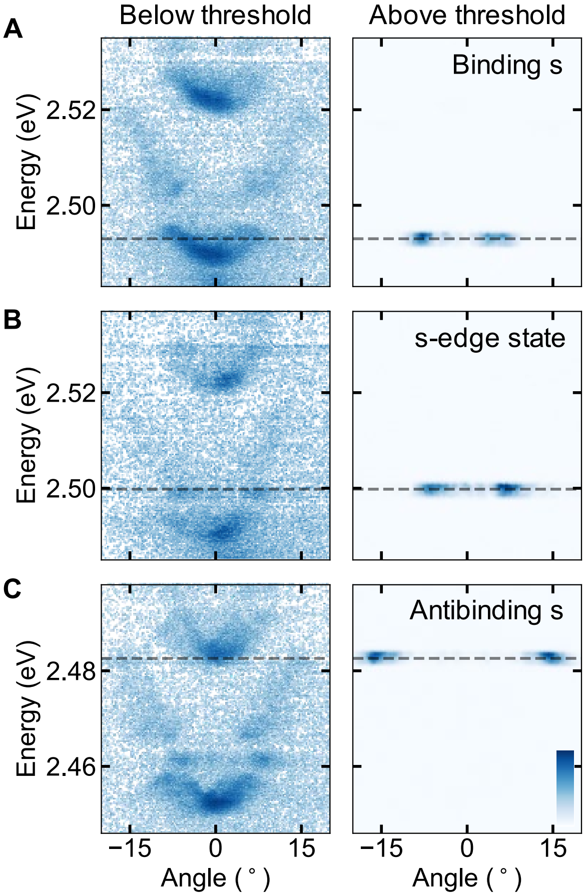

Fig. S3. Different lattice mode condensates in Fourier space. Angle-resolved photoluminescence below threshold (left panels) and above (right panels) in the binding s-mode (**A**), s-edge state (**B**) and antibinding s-mode (**C**). The angle of the PL is along the lattice structure.

Transition between selective condensation of s- and p-edge states

In the presented system, all the different lattice modes compete for the gain. In general, the favored state for polariton condensation is the one in resonance with the vibronic transition at around 2.51 eV. Depending on the in-situ tuning of the lattice detuning, it is possible to investigate a regime where a single mode condenses or one where the modes share the gain and multimode condensation takes place. This is displayed in the Supplementary Figure S4 which shows the selective single mode condensation of s- and p-edge states and the transition between them. By tuning (closing) the cavity the energy spectrum can be positioned such that the s-edge state (orange) is at an energy of 2.51 eV, aligning with the vibronic resonance and as a result condenses alone. Opening the cavity leads to a red shift of the spectrum and therefore the p-edge state (blue) also starts aligning with the vibronic resonance and starts condensing too. When the cavity opens even further the s-edge state shifts further away from the vibronic resonance and the p-edge state becomes the dominant single mode condensate. This also indicates that for achieving single-mode condensation for the higher orbital states it might be beneficial to red shift the lower orbital states

further away from the vibronic transition rather than having the higher state perfectly aligned with it.

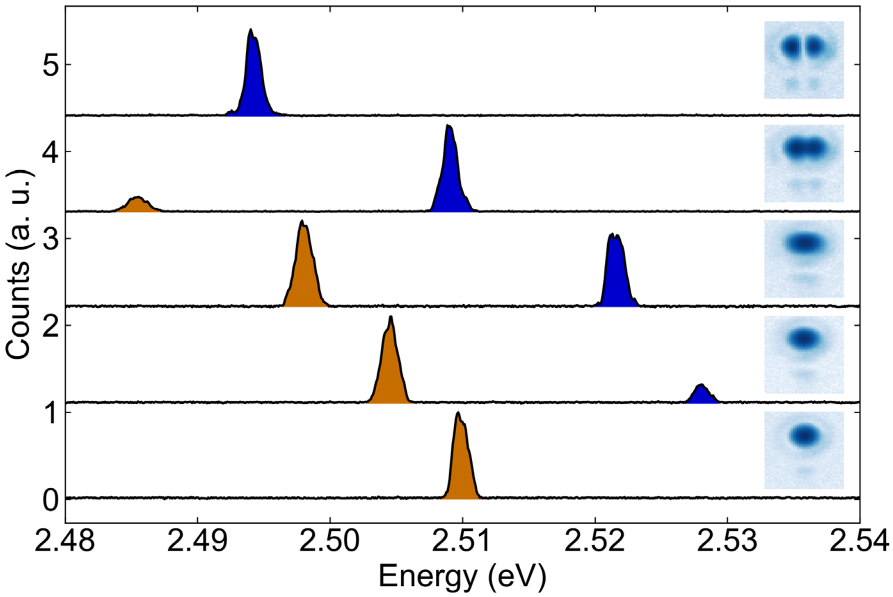

Fig. S4. Edge state tuning. Spectra and real space images of the cavity PL emission above threshold while pumping at the edge for different cavity lengths (s-edge state in orange, p-edge state in blue). Once a mode reaches around the 2.51 eV energy, aligning with the vibronic transition, it becomes the dominant single-mode condensate.

Extraction of the simulation parameters and estimation of effective coupling *J*

To use the previously described model, we first need to extract the value of the effective mass (m_{eff}) and the potential depth (V_{min}), that corresponds to a physical depth of 40 nm. Therefore, we measured the spectra of four pairs of coupled Gaussian deformations (Fig. S5A) that have the same site-to-site distance as the four bonds used for different SSH chains in the main experiment and consequently result in the same effective couplings *J*, respectively. We fitted the model to the doublet measurements, and we extracted $m_{\text{eff}} = 1.13 * 10^{-5} m_{\text{e}}$ with m_{e} being the electron mass and V_{min}= 80 meV. The simulated spectra, results of the fitting, are displayed in Fig. S5B.
To determine the effective couplings *J*, we used again the four experimental spectra of the coupled Gaussian deformations. Two coupled Gaussian deformations act as a polaritonic molecule, where evanescent coupling of the polariton wavefunctions leads to mode splitting. The resulting splitting is equal to twice the effective coupling *J* of the polaritonic molecule. For the weakest and strongest bonds used for the experiments, we measured a splitting equal to 3.9 meV and 18.3 meV, respectively (Fig. S5A).
Increasing the distance between the Gaussian deformations leads to a smaller overlap of the wavefunctions and therefore to an exponentially reduced effective coupling. This can be seen in Fig. S6 from the simulated mode splitting over a larger range of distances presented.

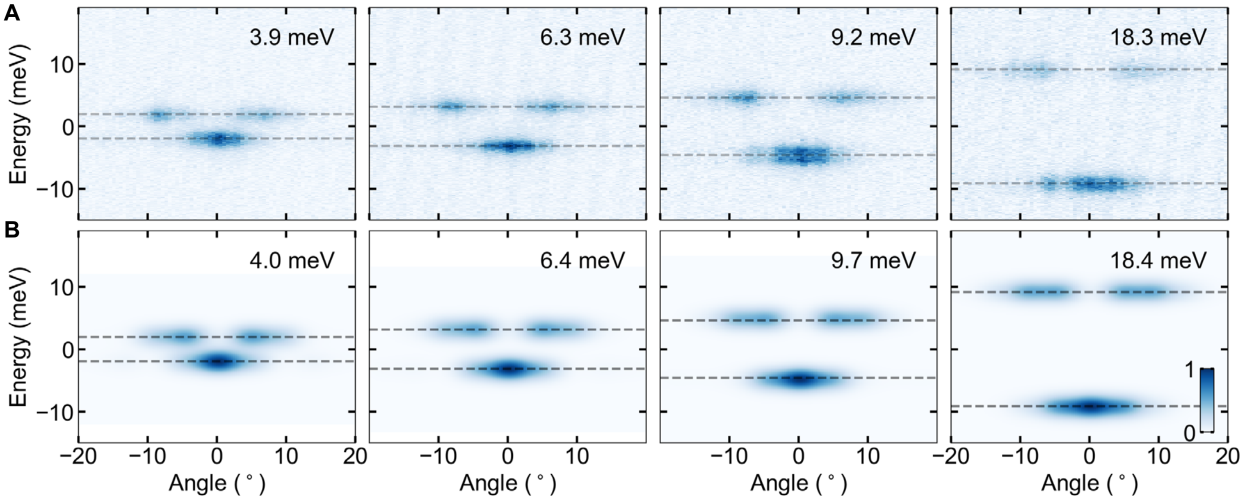

Fig. S5. Extraction of the simulation parameters from doublet measurements. (**A**) Angle-resolved photoluminescence of coupled Gaussian deformations with center-to-center distance decreasing from left to right: 1.44 µm, 1.26 µm, 1.08 µm, 0.72 µm. (**B**) Fitted angle-resolved spectra of doublets with the same spacings. The center of the y axes corresponds to the center of the gap. The dashed lines show the positions of the binding and antibinding s-like states extracted from the experimental data. The measured and calculated gaps are illustrated in the top right corner of each panel. All the images are normalized and described by the color bar inset in the bottom right part of last panel.

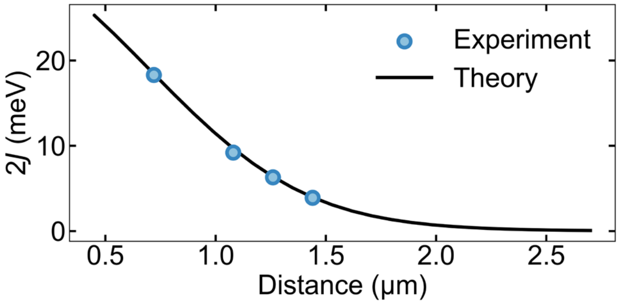

Fig. S6. Coupling strength as a function of distance for coupled Gaussian deformations. Measured (blue points) and calculated (black line) coupling strength over center-to-center distance between two Gaussian deformations.

<u>Band structure simulation and comparison to experiment</u>

After obtaining all the required parameters from the two coupled Gaussian deformations spectra, we use the model Hamiltonian to simulate the band structure of topologically trivial and non-trivial chains corresponding to different effective couplings for the weak bond of the SSH chain, presented in Fig. 4.

Both the experimental and simulated results are summarized in Fig. S7. The top panels (Fig. S7A) show the three experimentally measured band structures of Fig. 4, while the remaining six panels are numerical simulations of the trivial (Fig. S7B) and non-trivial (Fig. S7C) configurations of

structures with same number of sites and site-to-site spacing as the ones used for the experiment. All the simulated band structures are calculated such that the middle of the SSH gap resides at 2.51 eV, where polariton condensation is most efficient, as this corresponds to -200 meV vibronic shift from the exciton at 2.71 eV.

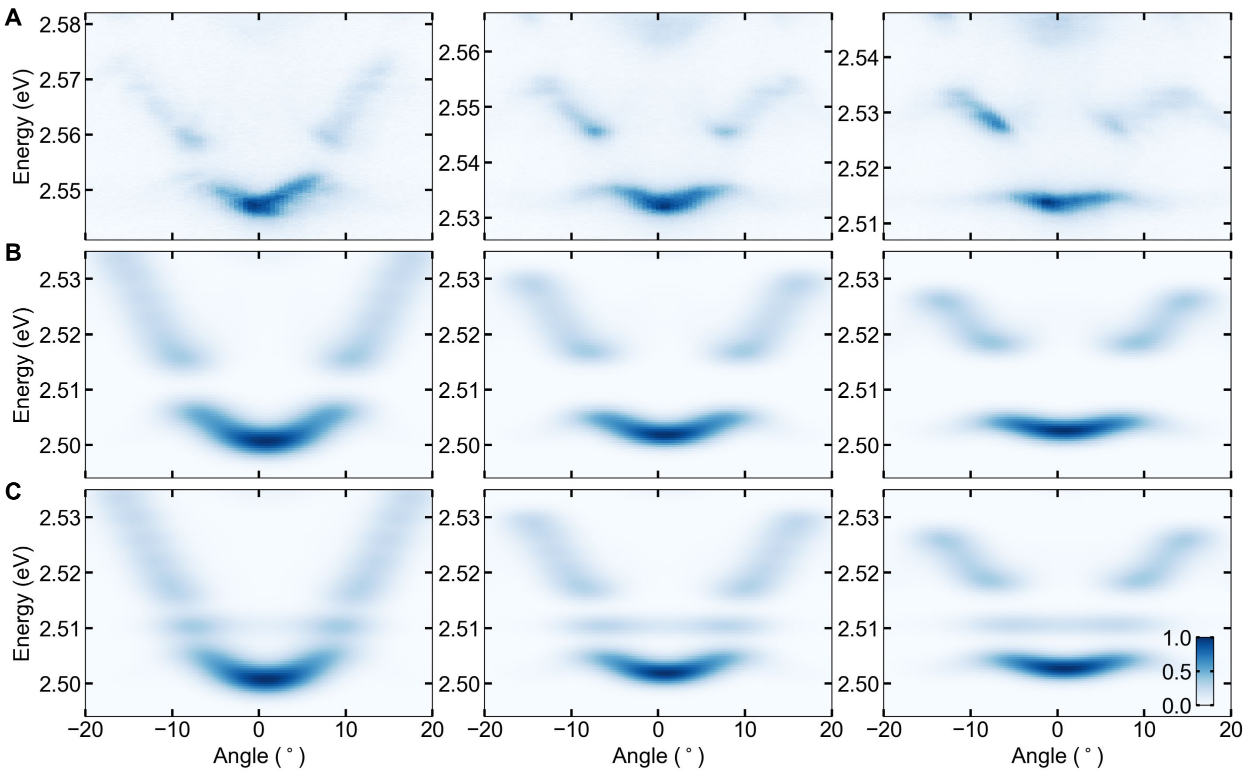

Fig. S7. Polariton band structure for the SSH chains. (**A**) Angle-resolved photoluminescence measurements of three trivial SSH structures with varying weak bonds. Calculated dispersions for trivial (**B**) and non-trivial (**C**) SSH chains. The calculated dispersions have the center of the SSH gap at 2.51 eV where the polariton condensation of the s-edge state occurs. Left, center and right panels correspond to center-to-center distance of the weak bond equal to 1.08 µm, 1.26 µm and 1.44 µm, respectively. All the images are normalized and described by the color bar inset in the bottom right part of last panel.

Impact of the tilt to the edge state position in the gap and edge state condensation

Sometimes in the experiment, the edge states are observed not perfectly in the middle of the SSH gap but somewhat closer to one of the band edges. A possible reason is tilt between the two cavity halves, which is a degree of freedom that we can control mechanically, but due to particles on the surfaces, not always with perfect precision. To assess the impact of this, we simulate the influence of an additional potential gradient on the energies of the edge states by calculating the energies of the lowest 14 eigenmodes using an effective Hamiltonian for 2D massive particles, utilizing the formalism described above. We focus on the non-trivial chain with center-to-center distance of the weak bond equal to 1.08 µm and varying potential gradients in the direction along the chain. The

energies of the modes forming the binding s-band, antibinding s-band and two edge states are illustrated in Fig. S8. The additional linear potential lifts the energetic degeneracy of the edge states and decreases the energy bandgap, with both becoming more pronounced with increasing tilt. Hence, the fact that we clearly observe in the experiment the edge states within the SSH gap allows us to put an upper limit of 0.2-0.4 meV/μm on the potential gradient, corresponding to 10-25 mdeg residual tilt. Fig. S9 shows selective condensation of both edges of a chain in the presence of ~4 meV tilt induced energy difference. These measurements were conducted for the chain with 0.72 μm strong and 1.26 μm weak bonds, resulting in a gradient of 0.3 meV/μm. It is obvious that the tilt does not impact the condensation into the edge states as long as the edge states are still located inside the energy gap (11 meV for this structure).

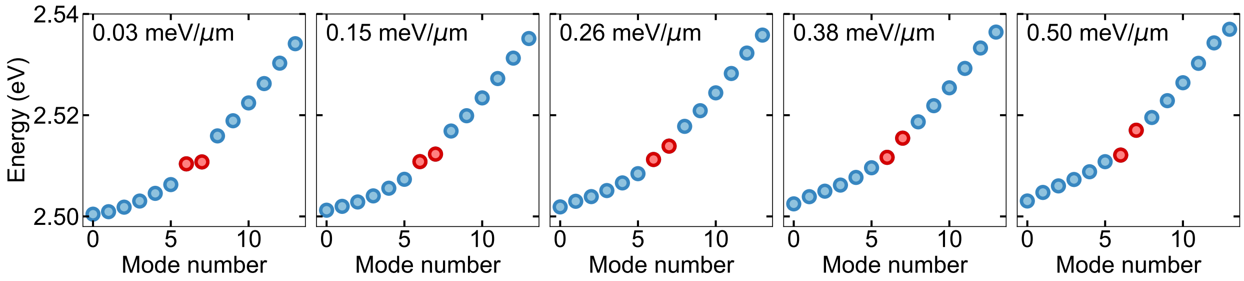

Fig. S8. Calculated eigenenergy spectrum for increasing potential gradient. Lowest eigenvalues of the SSH chain with an additional gradient potential. Blue points correspond to the modes forming binding and antibinding s-bands. Red points represent the edge states. Slopes of the potential are shown in the top left corners.

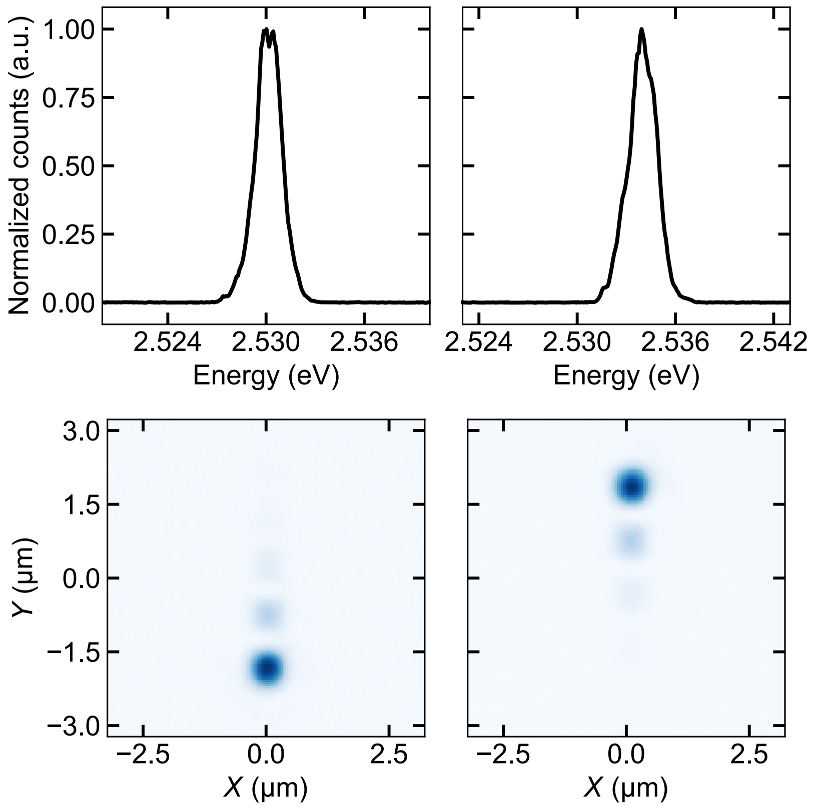

Fig. S9. Selective edge state condensation in the presence of tilt. Spectra (top) and real space images (bottom) of the cavity PL emission above threshold in both edges of the same SSH chain in the presence of tilt.

Simulating different levels of energetic disorder

Supplementary Figure S10 highlights the way that energetic disorder can affect our experiments of the SSH lattice. Fig. S10A shows that the variation in the potential depth of the different lattice sites shows a spread on the order of 3 nm. This corresponds to potential depth energy variation of less than 10 meV. Our simulations of the SSH chain with unequal well depths reveal a growing energy splitting between the two edge states with increasing imbalance in lattice site depths (Fig. S10B). However, the edge states are present in the gap even for the potential depth variation of 10 meV (Fig. S10C), suggesting that the topological effects in the system should still be unaffected.

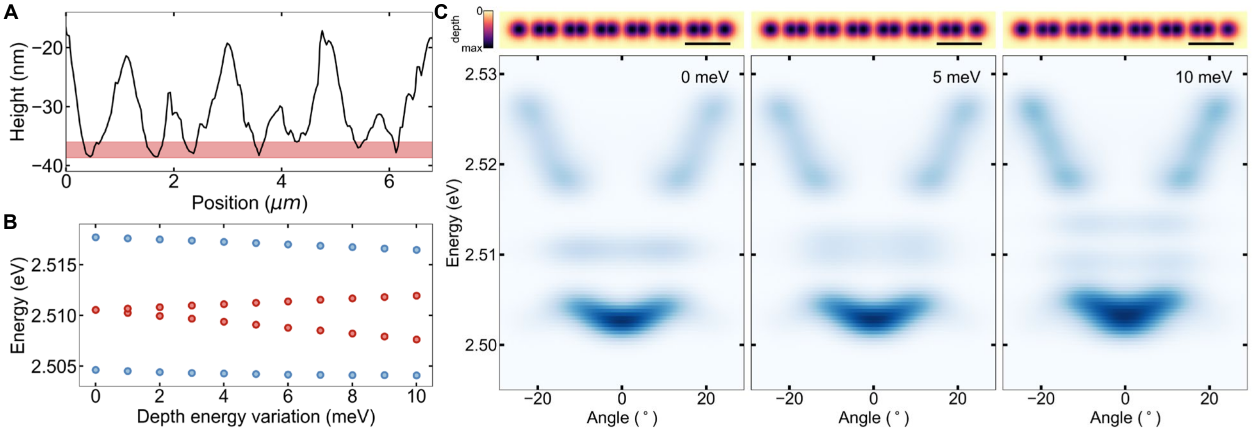

Fig. S10. Impact of energetic disorder in the SSH potential. (**A**) Profile cuts through the AFM image shown in Fig. 1, which was obtained after 2 years of measurements, with some contamination and defects that have occurred during and after the measurements. The highlighted red area has a height spread of 3 nm showing that the depths are equal or within that range. (**B**) Simulation of the energies of the edge states (red dots) and the states just below and above the SSH gap (blue dots) with increasing disorder. (**C**) Three simulated dispersions of the SSH chain for 0 meV, 5 meV and 10 meV disorder, respectively.

Simulation of the edge state localization and comparison to the experiment

We compare the experimental and the simulated edge state condensates for three different structures with different bandgaps, as displayed in Fig. 4. Fig. S11A shows the experimentally measured edge state condensates for three chains with different bandgaps (same as presented in Fig. 4 of the main text, included here for direct side-by-side comparison), while Fig. S11B shows the respective simulations. The localization length τ for the simulated data was extracted by fitting an exponential decay function to the local maxima.

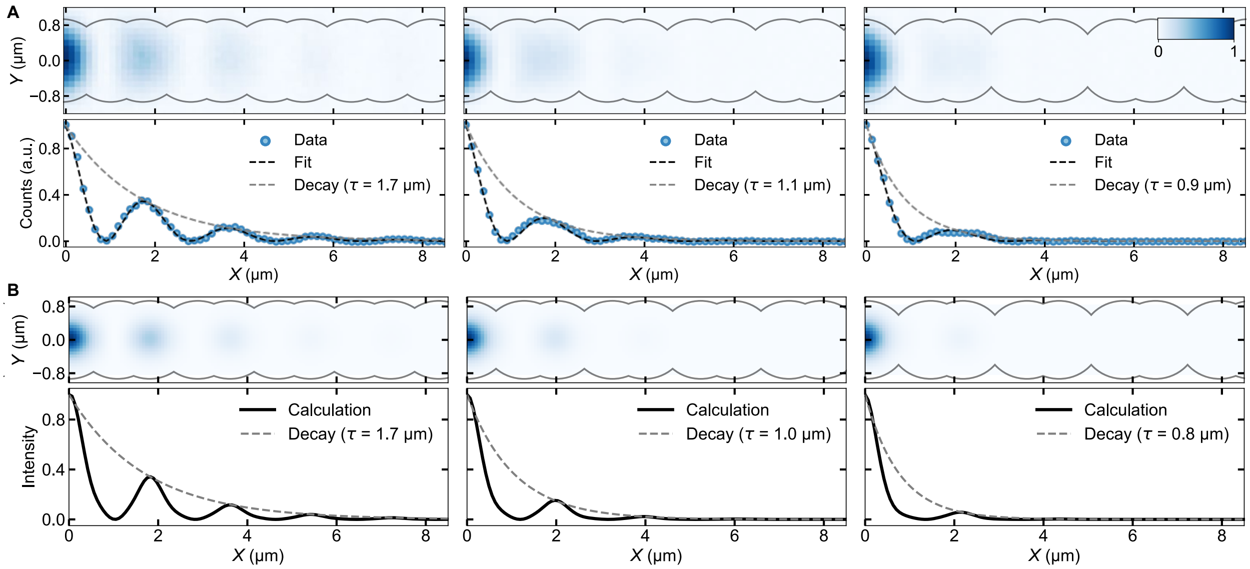

Fig. S11. Topological edge state engineering. Measured (**A**) and calculated (**B**) real space mode profiles (top panels) of the condensed s-band edge states for three non-trivial SSH chains and the fitted line profiles (bottom panels) with their respective calculated decay lengths. Left, center and right panels correspond to center-to-center distance of the weak bond equal to 1.08 μm, 1.26 μm and 1.44 μm respectively. All the images are normalized and described by the color bar inset in the top right panel.
